# Inferior vena cava point of care ultrasound: new perspectives in management of hyponatraemia

**DOI:** 10.1186/2036-7902-6-S2-A9

**Published:** 2014-08-27

**Authors:** P Pasquero, AV Taulaigo, S Albani, E Sitia, P Berchialla, L Borio, M Porta

**Affiliations:** 1 Department of Medical Sciences, A.O.U. Città della Salute e della Scienza, Torino, Italy; 2 Department of Clinical and Biological Sciences A.O.U. Città della Salute e della Scienza, Torino, Italy

## Background

Hyponatraemia is the most common electrolyte disorder in clinical practice, affecting 4% of patients presenting to the Emergency Department and up to 30% of patients in general medicine wards. It is associated with increased morbidity and mortality, therefore requiring prompt management. Current approach in defining aetiology of hypotonic hyponatraemia is based on extracellular fluid volume evaluation, which is potentially difficult, because of lack of highly sensible and specific clinical or laboratory tools. Evaluation of caval index through inferior vena cava point of care ultrasound could be helpful in defining intravascular volume status, as assessed in several conditions.

## Objective

Evaluate potential role of inferior vena cava ultrasonography to assess extracellular volume status in hyponatraemic patients.

## Patients and methods

Caval index was measured in 52 healthy blood donors and 21 normovolemic patients to define a normality range. Then caval index was measured in 16 patients affected by hypotonic hyponatraemia before starting any correction, hypervolemic patients were excluded. Each hyponatraemic patient has been also evaluated through standard clinical and laboratory tools by expert physicians, blinded to ultrasound measurements. Clinical and ultrasonographic evaluation were then compared.

## Results

Median Caval Index in normovolemic group was 0.35 with IQR (0.28-0.43). Caval Index in hyponatraemic patients showed a bimodal distribution, one peak corresponding to normovolemic median caval index and the other somewhat corresponding to median caval index of a hypovolemic population from a precedent study led by our group. Clinical and ultrasound evaluation were not concordant in 50% of patients, the former being more likely to miss hypovolemia.

## Conclusion

Inferior vena cava point of care ultrasound seems to be helpful in volume status evaluation, in conditions when evaluation is needed to be rapid and accurate, as hyponatraemia, being the consequent treatment different according to volume status.
